# Association between Benzodiazepine Use and Dementia: A Meta-Analysis

**DOI:** 10.1371/journal.pone.0127836

**Published:** 2015-05-27

**Authors:** GuoChao Zhong, Yi Wang, Yong Zhang, Yong Zhao

**Affiliations:** 1 The Second Student Office of Chongqing Medical University, Chongqing, China; 2 School of Public Health and Management, Chongqing Medical University, Chongqing, China; University of Groningen, NETHERLANDS

## Abstract

**Background:**

The association between long-term benzodiazepine use and risk of dementia remains controversial. Therefore, current study aimed to quantify this association, and to explore a potential dose–response pattern.

**Methods:**

We searched PubMed, Embase and the Cochrane Library through August 17, 2014. We included nested case-control or prospective cohort studies that provided risk estimates on the association of benzodiazepine use with risk of dementia, and a clear definition of status of benzodiazepine use. Overall effect size was calculated using a random-effects model.

**Findings:**

Six studies were eligible for inclusion, involving 11,891 dementia cases and 45,391 participants. Compared with never users, pooled adjusted risk ratios (RRs) for dementia were 1.49 (95% confidence interval (CI) 1.30–1.72) for ever users, 1.55 (95% CI 1.31–1.83) for recent users, and 1.55 (95% CI 1.17–2.03) for past users. The risk of dementia increased by 22% for every additional 20 defined daily dose per year (RR, 1.22, 95%CI 1.18–1.25). When we restricted our meta-analyses to unadjusted RRs, all initial significant associations persisted.

**Conclusions:**

Long-term benzodiazepine users have an increased risk of dementia compared with never users. However, findings from our study should be treated with caution due to limited studies and potential reverse causation. Large prospective cohort studies with long follow-up duration are warranted to confirm these findings.

## Introduction

As the number of older adults grows dramatically worldwide, dementia is regarded as a public health priority that has imposed an increased burden on our society in either financial or psychological respect. Nearly 35.6 million people were afflicted with dementia in 2012, and this number is expected to double by 2030 [1]. Benzodiazepines are a class of drugs acting on γ aminobutyric acid A receptors [2], and are commonly prescribed for the treatment of anxiety, insomnia and depression in the elderly across many countries [3–5]. The percentage of benzodiazepine use in subjects aged 65 years and over is 18.9% [5] and 15.0% [6] in Germany and Canada, respectively. In China, nearly one-third of patients with insomnia take benzodiazepines [7].

The guidelines recommend that the overall duration of benzodiazepine use should be limited to several weeks [8], but long-term use is still common [3, 9, 10]. Studies performed in patients aged 65 years and over reported a seven-year period of benzodiazepine use on average [11]. Several observational studies investigated the association between long-term benzodiazepine use and risk of dementia but presented mixed results. Some studies revealed an increased risk of dementia [12–16] among benzodiazepine users, whereas other studies revealed a decreased risk of dementia [17].

Meta-analysis is usually described as a quantitative statistical method that improves statistical power through pooling effect sizes from individual studies, and plays an important role in solving controversial issues in medicine. Therefore, we conducted a meta-analysis to quantify the relationship between long-term benzodiazepine use and dementia. Moreover, we explored a potential dose–response pattern between long-term benzodiazepine use and dementia.

## Materials and Methods

### 1. Search strategy

We performed this study following the PRISMA statement. Two researchers (G.C.Z. and Y.W.) independently performed an electronic search of PubMed, Embase and the Cochrane Library through August 17, 2014, with the following keywords: “dementia”, “Alzheimer's disease”, “Alzheimer disease”, “vascular dementia”, “benzodiazepines”, “benzodiazepine”, “hypnotics”, “hypnotic”, “sedatives” and “sedative”. We limited our searches to human subjects. The reference lists of identified studies and pertinent reviews were checked for identifying additional citations. No effort was attempted to obtain extra information by contacting the authors of included studies via e-mails.

### 2. Study selection

Nested case-control or prospective cohort studies that provided a clear definition of status of benzodiazepine use (ever use, recent use, or past use) and adjusted risk estimates on the association between benzodiazepine use and risk of dementia met our inclusion criteria. We excluded studies that compared recent users with a reference group that indiscriminately pooled both past and never users.

Two reviewers (G.C.Z. and Y.W.) independently performed study screening with a two-stage method. In the first stage, we read titles and abstracts to exclude obviously irrelevant studies. In the second stage, we read the full text to further exclude unrelated studies. Any discrepancy about eligibility of studies was resolved by discussion.

### 3. Data extraction

Two reviewers (G.C.Z. and Y.W.) independently extracted following information: last name of the first author, publication date, study location, mean age of participants at baseline, number of cases and participants, maximum follow-up duration, exposure assessment, outcome assessment, status of benzodiazepine use and corresponding definitions, diagnostic criteria, the crude risk estimates with 95% confidence interval (CI), the most fully adjusted risk estimates with 95% CI, and adjustment factors. Any disagreement was settled by discussion. When multiple reports derived from the same cohort, we extracted data from the report with the longest follow-up duration.

### 4. Statistical analysis and quality assessment

We used risk ratio (RR) to assess the association between benzodiazepine use and risk of dementia. Hazard ratio and odds ratio were directly regarded as RR because dementia is a relatively uncommon event [18, 19]. These primary effect sizes of interest are computed via logistic regression model or Cox proportional hazards model among included studies. We pooled adjusted RRs as well as crude RRs where possible to yield summary effect size through a random-effects model, which considered heterogeneity and consequently yielded more conservative pooled results compared to a fixed-effects model. The Hedges Q statistic was employed to qualitatively describe heterogeneity (statistical significance was set at *p*<0.10). The I^2^ statistic, representing the percentage of the variability in risk estimates that is caused by heterogeneity rather than chance [20], was employed to quantify heterogeneity [21]. Summary results were thought to be low heterogeneity if corresponding I^2^ <50%, moderate heterogeneity if 50%≦I2≦75%, and substantial heterogeneity if I^2^>75%. For two studies [15, 16] that reported risk estimates for recent and past users of benzodiazepines separately, we combined these stratum data using a random-effects model to approximate risk estimates for ever users. To evaluate the stability of pooled results and to identify potential sources of heterogeneity, we conducted sensitivity analyses for pooled adjusted RRs by means of omitting a single study in turn and using various exclusion criteria. We also repeated our meta-analyses through a fixed-effects model to evaluate the stability of our pooled results.

We explored a dose–response pattern between benzodiazepine use and dementia on the basis of the method previously proposed by Orsini and colleagues [22]. The independent variable was defined daily dose per year (defined daily dose, a dose unit for benzodiazepines, is referred to as “the assumed average maintenance dose per day for a drug used for its main indication in adults” [23]), and the dependent variable was the natural logarithm of the RRs. Using data on the defined daily dose per year, number of cases, person-year of follow-up, adjusted RR and corresponding 95% CI, we first calculated study-specific slope with log-linear dose-response model, and then combined them with a random-effects model to produce an overall slope. For two studies [14, 15] that provided cumulative benzodiazepine dose during follow-up, we divided cumulative dose by mean length of follow-up to obtain average defined daily dose per year. The midpoint of lower and upper boundaries was regarded as the assigned dose because benzodiazepine dose was reported as range in all available studies. If the highest range was open-ended, we assumed that it had the same width of the interval as the preceding range.

We employed the Newcastle–Ottawa quality assessment scale [24] to judge the methodological quality of included studies. This scale uses a star system to evaluate the quality of included studies on the basis of three broad perspectives: the selection of study cohort, the comparability of study cohort, and the ascertainment of outcome of interest, length of follow-up and loss to follow-up rate. After judging these three perspectives, a maximum of nine stars could be assigned to each study. A study with six or more stars was thought to be of high quality. We used Begg’s test [25] and Egger’s test [26] to test publication bias. STATA software (version12.0, StataCorp, College Station, TX) was used to perform data synthesis and analysis. Statistical significance level was set at *p*<0.05 under two-sided test unless otherwise specified.

## Results

### 1. Literature search

**Fig 1:**
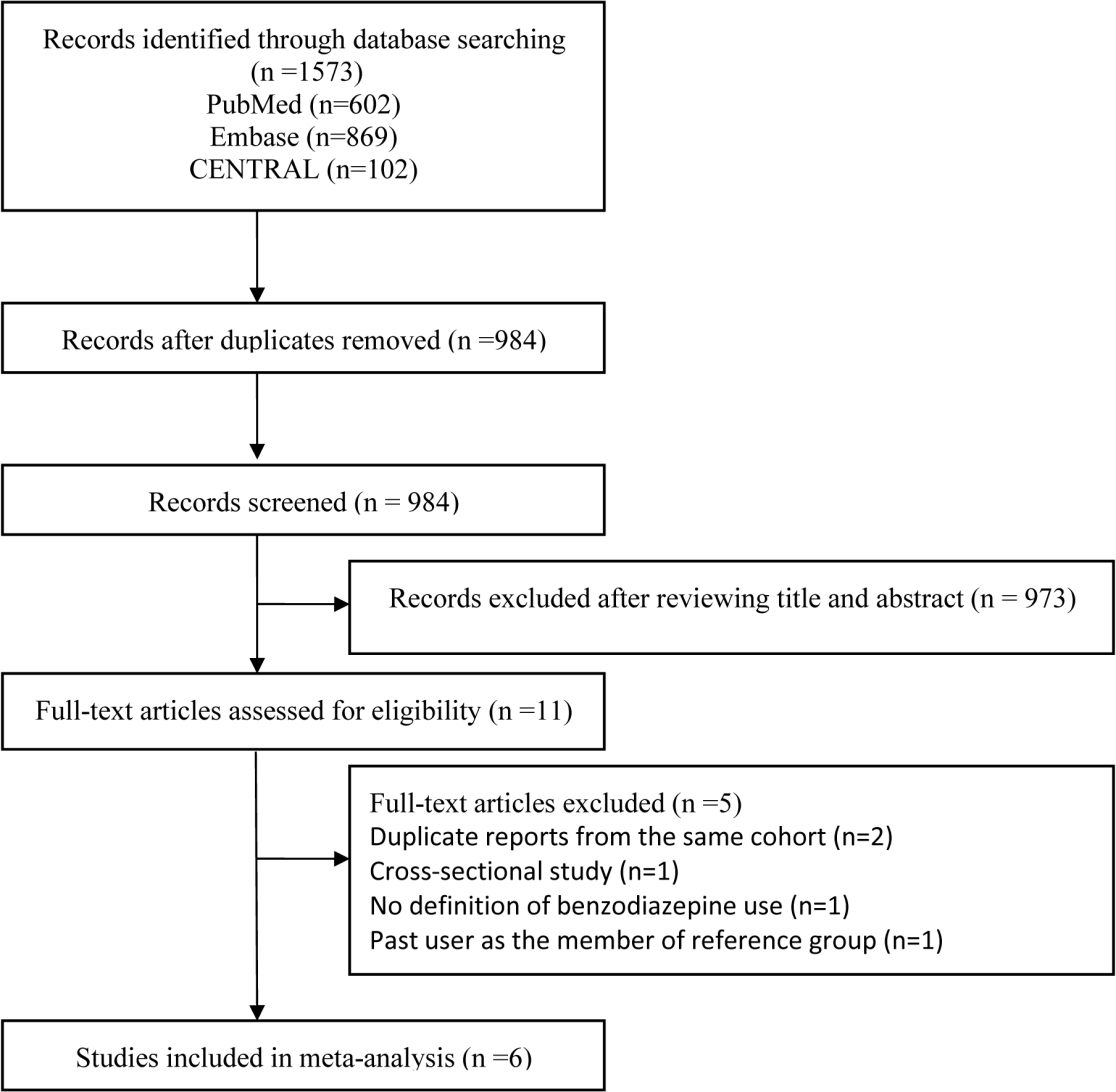
The flowchart of identifying relevant studies.

A sum of 1573 records (PubMed, n = 602; Embase, n = 869; CENTRAL, n = 102) were identified through our initial database search. After removing duplicates, 984 relevant records remained. Of these remained records, 978 were judged to be not eligible for the inclusion using our two-stage method. Thus, 6 studies were included in our review and analysis (Fig 1).

### 2. Study characteristics

The characteristics of included studies are presented in Table 1. These studies were published between 2002 [27] and 2013 [16]. The maximum follow-up duration ranged from 8 years [15, 27] to 25 years [12]. The sample size of included studies varied from 1063 [13] to 25,140 [14], with a total of 45,391 participants. The number of dementia cases varied from 93 [12] to 8,434 [14], with a sum of 11,891 dementia cases. Two studies [14, 15] utilized data from different subsets of the National Health Insurance Research Database in Taiwan. Five studies [12–14, 16, 27] separately provided RRs for recent and past users of benzodiazepines. Of these, two [13, 27] derived from the same cohort, but dementia cases were totally different, which made us treat them as two separate studies. One study [13] performed a cohort analysis, and additionally used a nested case-control analysis to check the robustness of their findings. Three studies [12, 13, 27] used the interview to document benzodiazepine exposure, two [14, 15] used the recorded prescription, and remaining one [16] used the claim record. The methods used to ascertain dementia included clinical diagnosis [12, 13, 27] and use of claim record [14, 15, 16]. For the quality assessment, all included studies obtained more than 6 stars, signifying high quality among them. Most of included studies adjusted confounders of age, anxiety and depression.

**Table 1:** Characteristics of 6 included studies regarding benzodiazepine use and dementia.

Source/study location	Cases/ SS	Age^a^ (y)	Follow-up^b^ (y)	Quality score	Diagnostic criteria	Exposure assessment	Outcome assessment	Definition of status of BZD use	Adjustment factors
Billioti de Gage [16], 2014; Canada	1796 8980	66.0	10	6	ICD-9	Claim record	Claim record	Recent use: first BZD claim <5 years before index date Past use: last BZD claim >5 years before index date	Anxiety, depression, psychotropic drugs
Gallacher [12], 2012; UK	93/ 1134	61.2	25	7	DSM-IV	Interview	Clinical diagnosis	Ever use: BZD use during follow-up period Recent use: first BZD claim <12 years before index date Past use: last BZD claim >12 years before index date	Age, anxiety, sleep disorders, angina, alcohol, cognitive function, education, ischemic heart disease, social class
Billioti de Gage [13], 2012; France (cohort analysis)	639/ 1063	78.2	15	8	DSM-III-R	Face-to-face interview	Clinical diagnosis	Ever use: BZD use during follow-up period	Age, sex, depression, alcohol, diabetes, education, hypertension, mini-mental state examination evolution between inclusion and 3 year follow-up visit, singleness, use of platelet inhibitors or oral anticoagulants
Billioti de Gage [13], 2012; France (nested case-control analysis)	467/ 2277	78.2	12	8	DSM-III-R	Face-to-face interview	Clinical diagnosis	Recent use: BZD use at the follow-up visit before index date but never before Past use: BZD use at least three visits before index date or earlier	Depression, alcohol, education, hypertension, singleness, use of platelet inhibitors or oral anticoagulant
Wu [14], 2011; China^c^	8434/25140	77.7	10	7	ICD-9	Recorded prescription	Claim record or inpatient record	Recent use^d^: first BZD claim <2 years before index date Past use^d^: last BZD claim >2 years before index date	Anxiety, depression, sleep disorders, alcohol, cerebrovascular disorders, diabetes, epilepsy, parkinsonism, hypertension, numbers of hospitalizations per year, psychosis-related disorders
Wu [15], 2009; China^c^	779/ 5405	75.6	8	8	ICD-9	Recorded prescription	Claim record	Ever use: BZD use during follow-up period	Anxiety, depression, alcohol, cerebrovascular disorder, diabetes, hypertension, psychotic-related disorder
Lagnaoui [27], 2002; France	150/ 3669	74.1	8	9	DSM-III-R	Face-to-face interview	Clinical diagnosis	Ever use: BZD use during follow-up period Recent use: first BZD claim <2 years before index date Past use: last BZD claim >2 years before index date	Age, sex, depression, alcohol, education, singleness, history of other psychiatric diseases

SS, sample size; BZD, benzodiazepine; ICD-9, International Classification of Diseases, Ninth Revision; DSM-IV, Diagnostic and Statistical Manual of Mental Disorders, fourth edition; DSM-III-R, Diagnostic and Statistical Manual of Mental Disorders, third edition Revised.

^a^Age refers to mean age of participants at baseline.

^b^Follow-up refers to maximum follow-up length.

^c^Using data of different subsets of National Health Insurance Research Database in Taiwan

^d^Original authors defined recent use and past use as first BZD claim <15 days and last BZD claim >15 days before index date, respectively. To keep homogenous with definitions of BZD use in other studies as much as possible, we redefined recent use and past use as first BZD claim <2 years and last BZD claim >2 years before index date, respectively. In this case, we pooled initial estimates to yield required estimates for “redefined" recent and past use through a random-effects model.

### 3. Ever use of benzodiazepines and risk of dementia

**Fig 2:**
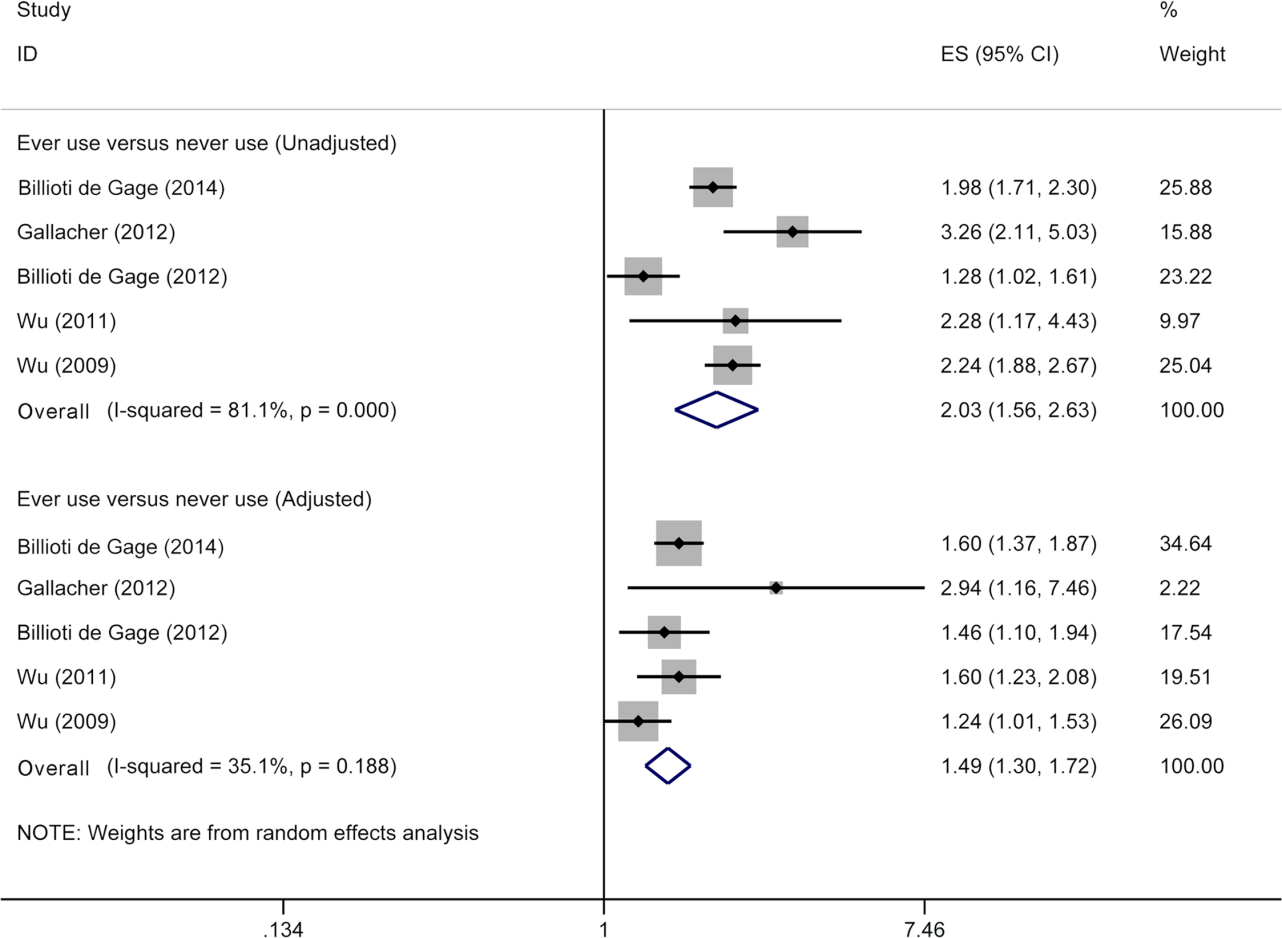
Meta-analysis on ever use of benzodiazepines and risk of dementia. The squares represent the risk estimate for each individual study, with the area reflecting the weight assigned to the study. The horizontal line across each square represents the 95% confidence interval. The diamond represents the summary risk estimate, with width representing 95% confidence interval.

Although six included studies all provided risk estimates associated with ever use of benzodiazepines, two of them [13, 27] derived from the same cohort. Thus, five studies [12–16] were included in meta-analysis for ever use of benzodiazepine and risk of dementia, involving 11,741 dementia cases and 41,722 subjects. When we pooled unadjusted RRs, ever users showed an increased risk of dementia (RR 2.03, 95% CI 1.56–2.63) (Fig 2) compared with never users. After adjustment for confounders, pooled RRs remained significant (RR 1.49, 95% CI 1.30–1.72) (Fig 2), with low heterogeneity (*p* = 0.19; I^2^ = 35.1%).

### 4. Recent use of benzodiazepines and risk of dementia

**Fig 3:**
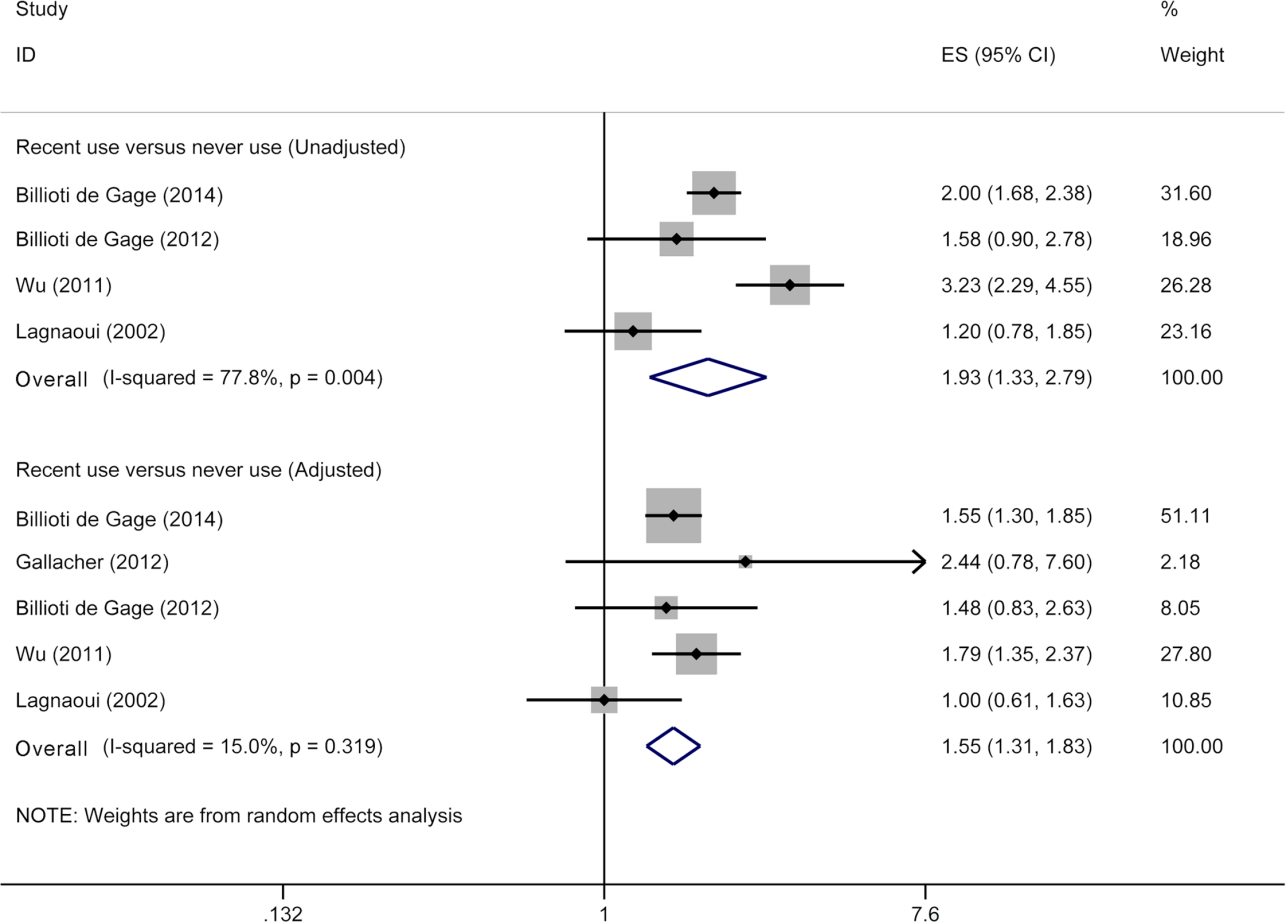
Meta-analysis on recent use of benzodiazepines and risk of dementia. The squares represent the risk estimate for each individual study, with the area reflecting the weight assigned to the study. The horizontal line across each square represents the 95% confidence interval. The diamond represents the summary risk estimate, with width representing 95% confidence interval.

Current meta-analysis for recent use of benzodiazepine and risk of dementia included five studies [12–14, 16, 27], involving 10,940 dementia cases and 41,200 participants. Recent users had a higher risk of dementia (RR 1.93, 95% CI 1.33–2.79) (Fig 3) than that for never users when we pooled unadjusted RRs. After adjustment for confounders, pooled RRs remained significant (RR 1.55, 95% CI 1.31–1.83) (Fig 3), with low heterogeneity (*p* = 0.32; I^2^ = 15.0%).

### 5. Past use of benzodiazepines and risk of dementia

**Fig 4:**
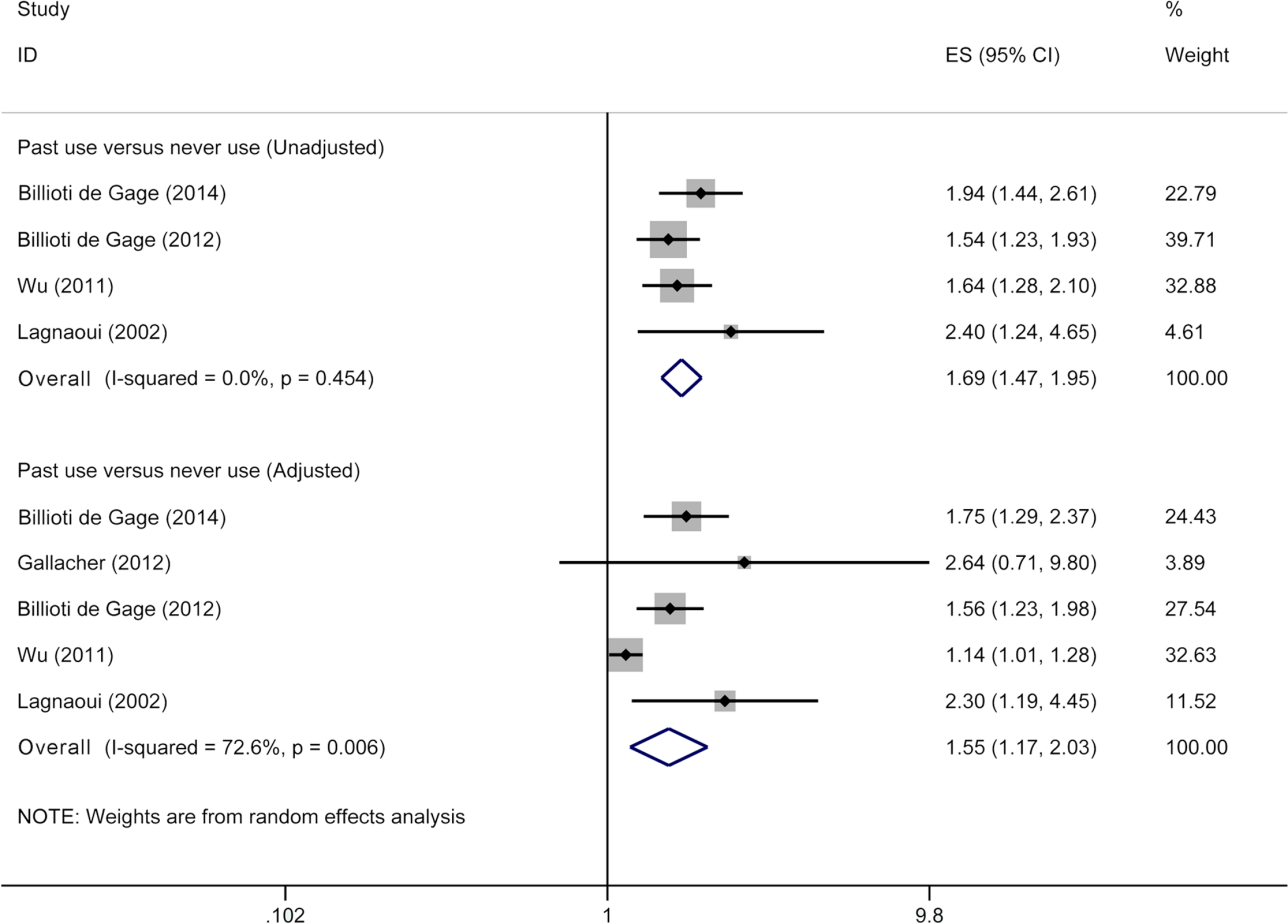
Meta-analysis on past use of benzodiazepines and risk of dementia. The squares represent the risk estimate for each individual study, with the area reflecting the weight assigned to the study. The horizontal line across each square represents the 95% confidence interval. The diamond represents the summary risk estimate, with width representing 95% confidence interval.

Our review included five studies [12–14, 16, 27] for past use of benzodiazepines and risk of dementia, involving 10,940 dementia cases and 41,200 participants. Past users had 1.69-fold higher risk of dementia (95% CI 1.47–1.95) (Fig 4) compared with never users when we pooled unadjusted RRs. After adjustment for confounders, pooled RRs remained significant (RR 1.55, 95% CI 1.17–2.03) (Fig 4), with moderate heterogeneity (*p*<0.01; I^2^ = 72.6%).

### 6. Sensitivity analyses

**Fig 5:**
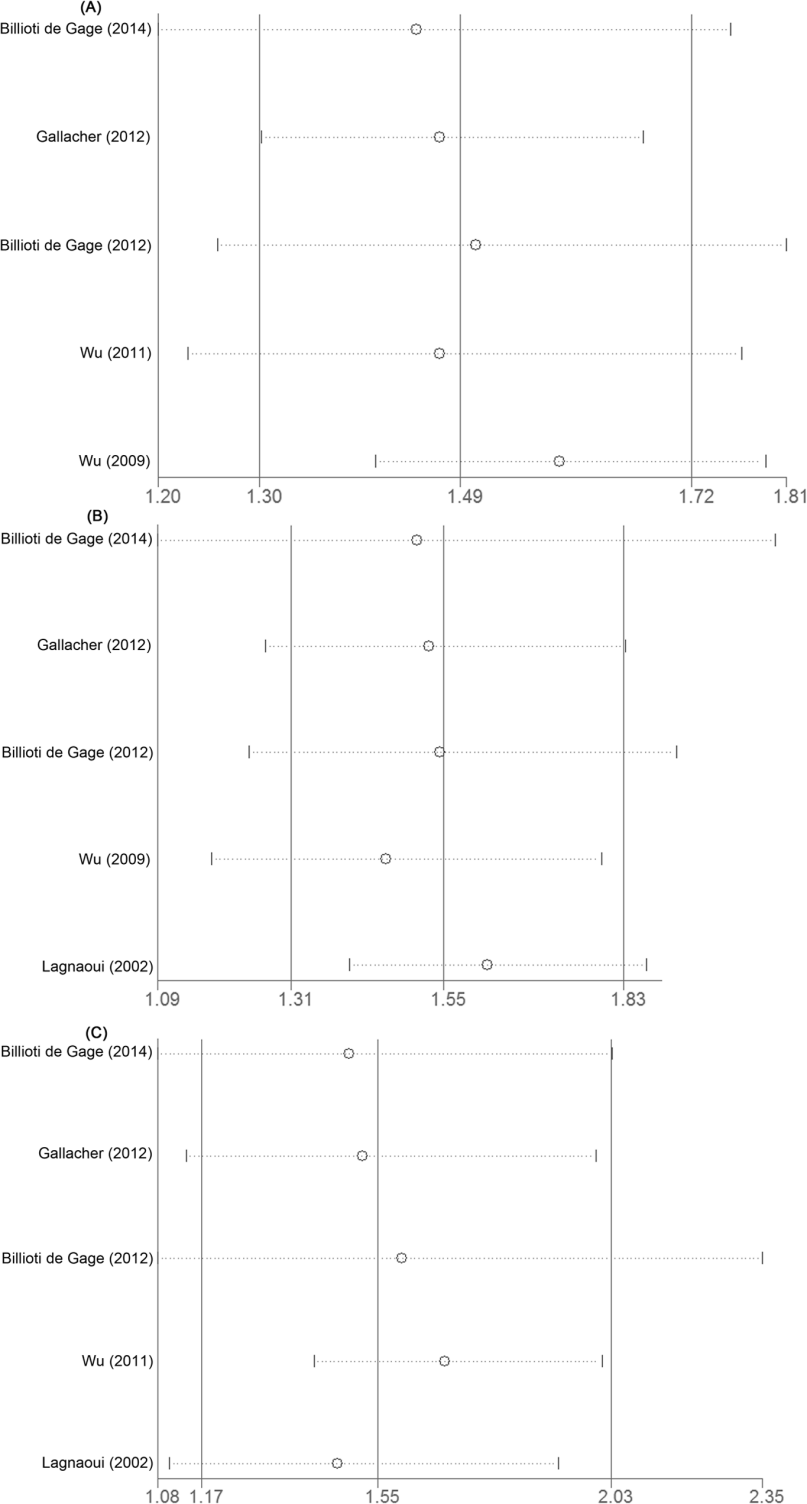
Sensitivity analysis: exclusion of a single study in turn. The study being cited on the left is the one being left out in each analysis. The circle represents the summary risk estimates after exclusion of a single study, and the corresponding dot line represents 95% confidence interval. The middle vertical solid line represents summary risk estimates of all included studies, and left and right vertical solid line represent lower limit and upper limit, respectively. Panel A, ever use versus never use; Panel B, recent use versus never use; Panel C, past use versus never use.

The results of sensitivity analyses are shown in Table 2 and Fig 5. Exclusion of a single study in turn did not change pooled RRs for the aforementioned associations. When we repeated our meta-analyses through a fixed-effects model, all initial significant associations persisted. For the association between past use of benzodiazepines and dementia, exclusion of one study [14] that conducted in the Asian population and had a sample size of more than 10,000 produced a RR of 1.69 (95% CI 1.41–2.02), with no evidence of heterogeneity (I^2^ = 0.0%).

**Table 2:** Sensitivity analyses of benzodiazepine use and dementia.

Categories	Ever use versus never use			Recent use versus never use			Past use versus never use		
	n	RR^a^ (95% CI)	I^2^ (%)	n	RR^a^(95% CI)	I^2^ (%)	n	RR^a^ (95% CI)	I^2^ (%)
*Statistical model*									
Random-effects model	5	1.49 (1.30–1.72)	35.1	5	1.55 (1.31–1.83)	15.0	5	1.55 (1.17–2.03)	72.6
Fixed-effects model	5	1.49 (1.35–1.66)	35.1	5	1.56 (1.35–1.79)	15.0	5	1.29 (1.16–1.42)	72.6
*Analyis within*									
Participants aged over 65 years at baseline	4	1.47 (1.30–1.67)	26.8	4	1.52 (1.27–1.84)	26.8	4	1.51 (1.14–2.00)	77.7
RRs with adjustment for anxiety	4	1.51 (1.25–1.81)	51.1	3	1.63 (1.40–1.89)	0.0	3	1.45 (0.97–2.17)	75.2
RRs with adjustment for depression	4	1.47 (1.30–1.67)	26.8	4	1.52 (1.27–1.84)	26.8	4	1.51 (1.14–2.00)	77.7
*Analysis except*									
Studies conducted in the Asian population	3	1.59 (1.38–1.82)	1.4	4	1.46 (1.18–1.80)	13.1	4	1.69 (1.41–2.02)	0.0
Men as participants	4	1.47 (1.30–1.67)	26.8	4	1.52 (1.27–1.84)	26.8	4	1.51 (1.14–2.00)	77.7
Sample size>10,000	4	1.47 (1.23–1.77)	48.7	4	1.46 (1.18–1.80)	13.1	4	1.69 (1.41–2.02)	0.0

RR, risk ratio; CI, confidence interval.

^a^Adjusted value.

### 7. Dose–response analysis

On the basis of only two studies [14, 15], we found a significant dose–response association between benzodiazepine use and dementia. The risk of dementia increased by 22% for a 20 defined daily dose per year increment in benzodiazepine dose (RR 1.22, 95% CI 1.18–1.25), with no evidence of heterogeneity (*p* = 0.32; I^2^ = 0.0%).

### 8. Publication bias

We did not find the evidence of publication bias for any association through Begg’s test and Egger’s test (all *p*>0.05).

## Discussion

### 1. Main findings

The association between long-term benzodiazepine use and risk of dementia has received significant attention but is still in debate. A most recent systematic review of observational studies (cohort and case-control studies) found that the long-term users of benzodiazepines have a 1.5- to 2-fold increased risk of developing dementia compared with never users [28]. On the basis of nested case-control and prospective cohort studies, our meta-analysis found that compared with never users, long-term benzodiazepine users (ever, recent and past users) were at an elevated risk of dementia when we pooled either unadjusted RRs or adjusted RRs, which was consistent with the findings of previous review [28].

The prodromal symptoms of dementia, including sleep disturbance, anxiety and depression [29–31], can occur around 10 years preceding a clinical diagnosis of dementia [32]. The presence of these psychiatric symptoms may motivate physicians to prescribe benzodiazepines. Thus, some researchers raise concerns that the observed association between benzodiazepine use and dementia may be due to confounding by indication and reverse causation [31, 33]. Nonetheless, the following facts possibly attenuate these concerns. First, it is found that the frequency of prodromes increases when dementia onset is approaching [32]. On the basis of this fact, the strength of the association for recent users should be stronger than that for past users if reverse causation is the case in the association of benzodiazepine use and dementia. However, our review observed similar risk estimates for recent and past users. Second, when we confined our analyses to RRs with adjustment for anxiety or depression, most but not all pooled results persisted. Third, a significant dose-response pattern was observed in the present study, although it was derived from limited numbers of included studies. Such finding supports a causal relationship between benzodiazepine use and dementia. Finally, our results are consistent with findings from an individual study [12] that had a mean follow-up of 22 years. The follow-up length in that study [12] was long enough to overcome the hypothesis of reverse causation.

The prevalence of psychotropic medication use among the elderly is high [34, 35], with a reported prevalence of up to 73% in subjects aged 65 years and over [34]. Use of psychotropic medications except benzodiazepines has been found to be associated with an increased risk of dementia [36, 37]. These facts provide a critical reminder that our findings might be biased by use of other psychotropic medication among included study population. There are two extreme conditions regarding this bias: one is that subjects in the exposure group concurrently use other psychotropic medications but those in the non-exposure group not, another is that subjects in the non-exposure group use other psychotropic medications but those in the exposure group not. The former condition results in an overestimated risk of dementia associated with benzodiazepine use, while the latter condition will underestimate that risk. Due to complex circumstances in the real world, unfortunately, we cannot ascertain the specific effect of use of other psychotropic medications on the magnitude and direction of the association between benzodiazepine use and dementia. Nonetheless, several included studies [13, 14, 27] treated benzodiazepine-related drugs, such as Z-drugs, as benzodiazepines, which produced a possibility of overestimating the risk of dementia in relation to benzodiazepine use.

Of included studies, ever use of benzodiazepines was consistently defined as benzodiazepine use during follow-up, namely use of benzodiazepines at least once before the index date. The consistent definition increased the reliability of our pooled results for the association between ever use of benzodiazepines and dementia. However, we observed that the cut-off points between recent use and past use among included studies varied from 2 years [27] to 12 years [12]. The selection of different cut-off points among included studies may be related to differences in the follow-up duration and interval between two follow-up visits. The different cut-off points represent inconsistent definitions of recent and past use of benzodiazepines among included studies. Under this condition, the exact definitions of recent and past use of benzodiazepines for the pooled results are uncertain. Consequently, the utility value of the findings associated with recent and past use of benzodiazepines may be affected to some extent. Nonetheless, our findings regarding recent and past use of benzodiazepines may provide an important implication that stopping use of benzodiazepines cannot significantly reduce the risk of developing dementia.

In this meta-analysis, we calculated summary RRs from unadjusted and adjusted data. Interestingly, a significantly positive association of benzodiazepine use with risk of dementia was consistently observed in both conditions. Furthermore, on the basis of *p* values for the difference between two conditions calculated from meta-regression (data not shown), we found that there was no significant difference in magnitude of the pooled effect size between two conditions. Therefore, pooled results from unadjusted and adjusted risk estimates resulted in virtually identical interpretation for the association of benzodiazepine use with risk of dementia. Consequently, calculation of unadjusted and adjusted summary RRs makes our findings more convincing rather than more obscuring.

### 2. Source of heterogeneity

In the current review, we found the evidence of moderate heterogeneity for pooled adjusted RRs on past use of benzodiazepines and dementia. As revealed by our sensitivity analyses, one study [14] that conducted in the Asian population and had a large sample size probably accounted for the observed heterogeneity. Indeed, racial differences in the prevalence and incidence of dementia have been reported [38, 39]. Epidemiological studies with large sample size are less driven by confounding effect and chance error, consequently producing more precise risk estimates.

### 3. Limitations

Our review has several limitations. First, inclusion of a small number of studies did not permit us to investigate the potential effect modifiers for the association of benzodiazepine use and dementia through subgroup analyses. For example, Alzheimer’s disease and vascular dementia are the main subtypes of dementia, and they have different incidence and etiology. However, we could not confirm whether the risk of Alzheimer’s disease is different from that of vascular dementia due to limited numbers of included studies. Second, although there was no evidence of publication bias, we could not completely rule it out because Begg’s test and Egger’s test have limited statistical power when there are relatively small included studies. Third, we observed moderate heterogeneity for pooled results on past use of benzodiazepines and dementia. Such heterogeneity raised concerns about the reliability of pooled results. Nevertheless, we were capable of identifying the source of heterogeneity through our sensitivity analyses. Finally, our pooled results might be subject to the uncontrolled or residual confounding, although we used the most fully adjusted risk estimates. Nevertheless, when we pooled unadjusted RRs, the increased risk of dementia in benzodiazepine users remained significant.

### 4. Implication for clinical practice

Prevention of dementia is critical under the condition that the pharmaceutical therapies for dementia fail to function well against disease progression [40]. Long-term benzodiazepine use has been linked with increased risks of injurious falls [41] and hip fracture [42]. On the basis of limited studies, we observed that long-term benzodiazepine use was associated with an increased risk of dementia. If these observed associations are causal, for reducing possible adverse reactions, ideally, medical practitioners should limit benzodiazepine use to several weeks as recommended by international guidelines [8]. However, in practice, making such decision is difficult because apprehension regarding the possibility of rebound anxiety and insomnia after discontinued use of benzodiazepine appears to favor the long-term use. Thus, physicians should carefully weigh the anticipated benefits of long-term benzodiazepine use against the risks involved.

## Conclusions

On the basis of either unadjusted or adjusted risk estimates, our study consistently indicates that long-term benzodiazepine use is associated with an increased risk of dementia. Due to limited studies, especially dose-response analysis, and potential reverse causation, these findings should be treated with caution. Large prospective cohort studies with long follow-up duration are needed to confirm whether the association between long-term benzodiazepine use and increased risk of dementia is causal. If confirmed, long-term benzodiazepine use should be considered as a critical public health issue in the context of the widespread use of benzodiazepines and the huge burden of dementia across many countries.

## Supporting Information

S1 Checklist PRISMA checklist. (DOC)

S1 Dataset Original data associated with the present study. (XLS)

## References

[1] World Health Organization. Dementia: a public health priority World Health Organization; 2012.

[2] Dell'osso B, Lader M. Do benzodiazepines still deserve a major role in the treatment of psychiatric disorders? A critical reappraisal. European psychiatry: the journal of the Association of European Psychiatrists. 2013;28(1):7–20. 22521806 10.1016/j.eurpsy.2011.11.003

[3] Ashton H. The diagnosis and management of benzodiazepine dependence. Current opinion in psychiatry. 2005;18(3):249–55. 16639148 10.1097/01.yco.0000165594.60434.84

[4] Shorr RI, Robin DW. Rational use of benzodiazepines in the elderly. Drugs & aging. 1994;4(1):9–20. 7907503 10.2165/00002512-199404010-00002

[5] Linden M, Bar T, Helmchen H. Prevalence and appropriateness of psychotropic drug use in old age: results from the Berlin Aging Study (BASE). International psychogeriatrics / IPA. 2004;16(4):461–80. 15715361 10.1017/s1041610204000420

[6] Mamdani M, Rapoport M, Shulman KI, Herrmann N, Rochon PA. Mental health-related drug utilization among older adults: prevalence, trends, and costs. The American journal of geriatric psychiatry: official journal of the American Association for Geriatric Psychiatry. 2005;13(10):892–900. 16223968 10.1176/appi.ajgp.13.10.892

[7] Xiang YT, Ma X, Cai ZJ, Li SR, Xiang YQ, Guo HL, et al The prevalence of insomnia, its sociodemographic and clinical correlates, and treatment in rural and urban regions of Beijing, China: a general population-based survey. Sleep. 2008;31(12):1655–62. 19090321 10.1093/sleep/31.12.1655 PMC2603488

[8] European Medicines Agency. Summary of Product Characteristics for Benzodiazepines as Anxiolytics or Hypnotics. 1994.

[9] Griffiths RR, Weerts EM. Benzodiazepine self-administration in humans and laboratory animals—implications for problems of long-term use and abuse. Psychopharmacology. 1997;134(1):1–37. 9399364 10.1007/s002130050422

[10] Vinkers CH, Olivier B. Mechanisms Underlying Tolerance after Long-Term Benzodiazepine Use: A Future for Subtype-Selective GABA(A) Receptor Modulators? Advances in pharmacological sciences. 2012;2012:416864 10.1155/2012/416864 22536226 PMC3321276

[11] Puustinen J, Nurminen J, Kukola M, Vahlberg T, Laine K, Kivela SL. Associations between use of benzodiazepines or related drugs and health, physical abilities and cognitive function: a non-randomised clinical study in the elderly. Drugs & aging. 2007;24(12):1045–59. 18020536 10.2165/00002512-200724120-00007

[12] Gallacher J, Elwood P, Pickering J, Bayer A, Fish M, Ben-Shlomo Y. Benzodiazepine use and risk of dementia: evidence from the Caerphilly Prospective Study (CaPS). Journal of epidemiology and community health. 2012;66(10):869–73. 10.1136/jech-2011-200314 22034632

[13] Billioti de Gage S, Begaud B, Bazin F, Verdoux H, Dartigues JF, Peres K, et al Benzodiazepine use and risk of dementia: prospective population based study. BMJ (Clinical research ed). 2012;345:e6231 10.1136/bmj.e6231 23045258 PMC3460255

[14] Wu CS, Ting TT, Wang SC, Chang IS, Lin KM. Effect of benzodiazepine discontinuation on dementia risk. The American journal of geriatric psychiatry: official journal of the American Association for Geriatric Psychiatry. 2011;19(2):151–9. 10.1097/JGP.0b013e3181e049ca 20808131

[15] Wu CS, Wang SC, Chang IS, Lin KM. The association between dementia and long-term use of benzodiazepine in the elderly: nested case-control study using claims data. The American journal of geriatric psychiatry: official journal of the American Association for Geriatric Psychiatry. 2009;17(7):614–20. 10.1097/JGP.0b013e3181a65210 19546656

[16] Billioti de Gage S, Moride Y, Ducruet T, Kurth T, Verdoux H, Tournier M, et al Benzodiazepine use and risk of Alzheimer's disease: case-control study. BMJ (Clinical research ed). 2014;349:g5205. 10.1136/bmj.g5205 PMC4159609 25208536

[17] Fastbom J, Forsell Y, Winblad B. Benzodiazepines may have protective effects against Alzheimer disease. Alzheimer disease and associated disorders. 1998;12(1):14–7. 9539405 10.1097/00002093-199803000-00002

[18] Greenland S. Quantitative methods in the review of epidemiologic literature. Epidemiologic reviews. 1987;9:1–30. 3678409 10.1093/oxfordjournals.epirev.a036298

[19] Dong JY, Zhang YH, Qin LQ. Erectile dysfunction and risk of cardiovascular disease: meta-analysis of prospective cohort studies. Journal of the American College of Cardiology. 2011;58(13):1378–85. 10.1016/j.jacc.2011.06.024 21920268

[20] Higgins JP, Thompson SG. Quantifying heterogeneity in a meta-analysis. Statistics in medicine. 2002;21(11):1539–58. 12111919 10.1002/sim.1186

[21] Higgins JP, Thompson SG, Deeks JJ, Altman DG. Measuring inconsistency in meta-analyses. BMJ (Clinical research ed). 2003;327(7414):557–60. 12958120 10.1136/bmj.327.7414.557 PMC192859

[22] Orsini N, Bellocco R, Greenland S. Generalized least squares for trend estimation of summarized dose–response data. The Stata Journal. 2006;6(1):40–57.

[23] World Health Organization. World Health Organization Collaborating Centre for Drug Statistics Methodology: Guidelines for ATC Classification and DDD Assignment. Oslo, Norway: WHO 2000.

[24] Wells G, Shea B, O’connell D, Peterson J, Welch V, Losos M, et al. The Newcastle-Ottawa Scale (NOS) for assessing the quality of nonrandomised studies in meta-analyses. 2000.

[25] Begg CB, Mazumdar M. Operating characteristics of a rank correlation test for publication bias. Biometrics. 1994;50(4):1088–101. 7786990

[26] Egger M, Davey Smith G, Schneider M, Minder C. Bias in meta-analysis detected by a simple, graphical test. BMJ (Clinical research ed). 1997;315(7109):629–34. 9310563 10.1136/bmj.315.7109.629 PMC2127453

[27] Lagnaoui R, Begaud B, Moore N, Chaslerie A, Fourrier A, Letenneur L, et al Benzodiazepine use and risk of dementia: a nested case-control study. Journal of clinical epidemiology. 2002;55(3):314–8. 11864804 10.1016/s0895-4356(01)00453-x

[28] Billioti de Gage S, Pariente A, Begaud B. Is there really a link between benzodiazepine use and the risk of dementia? Expert opinion on drug safety. 2015:1–15. 10.1517/14740338.2015.1014796 25691075

[29] Bennett S, Thomas AJ. Depression and dementia: Cause, consequence or coincidence? Maturitas. 2014. 10.1016/j.maturitas.2014.05.009 24931304

[30] Mirza SS, de Bruijn RF, Direk N, Hofman A, Koudstaal PJ, Ikram MA, et al. Depressive symptoms predict incident dementia during short- but not long-term follow-up period. Alzheimer's & dementia: the journal of the Alzheimer's Association. 2014. 10.1016/j.jalz.2013.10.006 24530024

[31] Bocti C, Roy-Desruisseaux J, Roberge P. Research paper most likely shows that benzodiazepines are used to treat early symptoms of dementia. BMJ (Clinical research ed). 2012;345:e7986; author reply e93. 10.1136/bmj.e7986 23183062

[32] Amieva H, Le Goff M, Millet X, Orgogozo JM, Peres K, Barberger-Gateau P, et al Prodromal Alzheimer's disease: successive emergence of the clinical symptoms. Annals of neurology. 2008;64(5):492–8. 10.1002/ana.21509 19067364

[33] Barbui C, Gastaldon C, Cipriani A. Benzodiazepines and risk of dementia: true association or reverse causation? Epidemiology and psychiatric sciences. 2013;22(4):307–8. 10.1017/S2045796013000358 23823009 PMC8367345

[34] Petek Ster M, Cedilnik Gorup E. Psychotropic medication use among elderly nursing home residents in Slovenia: cross-sectional study. Croatian medical journal. 2011;52(1):16–24. 21328716 10.3325/cmj.2011.52.16 PMC3046495

[35] Lesen E, Carlsten A, Skoog I, Waern M, Petzold M, Borjesson-Hanson A. Psychotropic drug use in relation to mental disorders and institutionalization among 95-year-olds: a population-based study. International psychogeriatrics / IPA. 2011;23(8):1270–7. 10.1017/S1041610211000524 21447258

[36] Hsiao F-Y, Peng L-N, Lin M-H, Chen L-K. Dose-Responsive Effect of Psychotropic Drug Use and Subsequent Dementia: A Nationwide Propensity Score Matched Case-Control Study in Taiwan. Journal of the American Medical Directors Association. 2014. 10.1016/j.jamda.2014.02.009 24685407

[37] Chen PL, Lee WJ, Sun WZ, Oyang YJ, Fuh JL. Risk of dementia in patients with insomnia and long-term use of hypnotics: a population-based retrospective cohort study. PloS one. 2012;7(11):e49113 10.1371/journal.pone.0049113 23145088 PMC3492301

[38] Parikh NM, Morgan RO, Kunik ME, Chen H, Aparasu RR, Yadav RK, et al Risk factors for dementia in patients over 65 with diabetes. International journal of geriatric psychiatry. 2011;26(7):749–57. 10.1002/gps.2604 20891020

[39] Froehlich TE, Bogardus ST Jr., Inouye SK. Dementia and race: are there differences between African Americans and Caucasians? Journal of the American Geriatrics Society. 2001;49(4):477–84. 11347796 10.1046/j.1532-5415.2001.49096.x

[40] Smith AD, Yaffe K. Dementia (including Alzheimer's disease) can be prevented: statement supported by international experts. Journal of Alzheimer's disease: JAD. 2014;38(4):699–703. 10.3233/JAD-132372 24326609

[41] Pariente A, Dartigues JF, Benichou J, Letenneur L, Moore N, Fourrier-Reglat A. Benzodiazepines and injurious falls in community dwelling elders. Drugs & aging. 2008;25(1):61–70. 18184030 10.2165/00002512-200825010-00007

[42] Ray WA, Griffin MR, Downey W. Benzodiazepines of long and short elimination half-life and the risk of hip fracture. JAMA: the journal of the American Medical Association. 1989;262(23):3303–7. 2573741

